# Developing a novel FRET assay, targeting the binding between Antizyme-AZIN

**DOI:** 10.1038/s41598-019-40929-4

**Published:** 2019-03-15

**Authors:** Aram Ghalali, James M. Rice, Amanda Kusztos, Finith E. Jernigan, Bruce R. Zetter, Michael S. Rogers

**Affiliations:** 1 Vascular Biology Program and Department of Surgery, Boston Children’s Hospital, Harvard Medical School, Boston, MA USA; 2 Center for Drug Discovery and Translational Research, Beth Israel Deaconess Medical Center, Harvard Medical School, Boston, MA USA; 3 Present Address: Silicon Therapeutics, Boston, MA USA; 4 Present Address: Department of Infectious Disease, Brigham and Women’s Hospital, Harvard Medical School, Boston, MA USA

## Abstract

Antizyme inhibitor (AZIN) stimulates cell proliferation by binding to and sequestering the cell cycle suppressor antizyme. Despite the important role of the antizyme-AZIN protein-protein interaction (PPI) in cell cycle regulation, there are no assays for directly measuring the binding of AZIN to antizyme that are amenable to high throughput screening. To address this problem, we developed and validated a novel antizyme-AZIN intramolecular FRET sensor using clover and mRuby2 fluorescent proteins. By introducing alanine mutations in the AZIN protein, we used this sensor to probe the PPI for key residues governing the binding interaction. We found that like many PPIs, the energy of the antizyme-AZIN binding interaction is distributed across many amino acid residues; mutation of individual residues did not have a significant effect on disrupting the PPI. We also examined the interaction between Clover-AZIN and antizyme-mRuby2 in cells. Evidence of a direct interaction between Clover-AZIN and antizyme-mRuby2 was observed within cells, validating the use of this FRET sensor for probing intracellular antizyme-AZIN PPI. In conclusion, we have developed and optimized a FRET sensor which can be adapted for high throughput screening of either *in vitro* or intracellular activity.

## Introduction

Antizyme is a well-characterized tumor suppressor that facilitates the proteasomal degradation of several growth promoting molecules including ornithine decarboxylase (ODC)^1^, Cyclin D1^2^, SMAD1^3^, and the Aurora kinase A^4^, and is important for normal cell cycle progression^5^. In addition to inducing ODC degradation, antizyme also inhibits ODC enzymatic activity and because ODC is the rate-limiting step in polyamine synthesis, antizyme expression also dampens intracellular polyamine levels in late G1 phase of the cell cycle^6^. Each of these effects contributes to antizyme-mediated restraint of cell proliferation and to its tumor-suppressor function^7^; loss or inactivation of antizyme leads to unrestrained cell proliferation^8^.

An endogenous antizyme inhibitor (AZIN) protein binds to antizyme and blocks its activity^9^. *AZIN1* gene expression is increased in various cancers including gastric, prostate, lung, liver, and ovary^9–11^. Furthermore, AZIN silencing leads to a reduction in tumor cell proliferation and tumor growth in model systems^12^, demonstrating a role for AZIN as a positive modulator of cancer cell growth. We further anticipate that agents that interfere with AZIN binding to antizyme could restore antizyme activity and repress cell growth in cancer and other proliferative diseases. Despite increasing recognition of the role of AZIN in cancer, no small molecule AZIN antagonists or assays for their development currently exist.

Here, we have developed a novel Förster resonance energy transfer (FRET) assay that will identify molecules that inhibit AZIN-antizyme binding, thereby releasing antizyme to inhibit cancer cell growth. The assay has been optimized and adapted for use in the molecular screening of small molecule libraries. This assay is also validated to measure the AZIN-antizyme interaction both *in vitro* and *in vivo*. We used this sensor in alanine screening mutagenesis to identify structural elements and potential key residues that mediate AZIN-antizyme binding.

## Results

### A novel FRET sensor can measure antizyme:AZIN interaction ***in vitro*** and ***in vivo***

**Figure 1:**
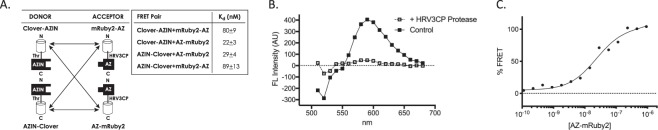
Design and Validation of a FRET based AZ-AZI protein-protein interaction sensor. (**A**) Four FRET fusion proteins were created including a GFP tagged AZIN protein (C and N terminal) and a mRuby2 tagged antizyme (AZ) protein (C and N terminal). The Kd of each interaction is shown. (**B**) The fluorescent difference spectra from the Clover-AZIN:AZ-mRuby2 FRET pair (ex 485) before and after cleavage of AZ-mRuby2 fusion protein with HRV 3 C protease. Difference spectra were calculated by subtracting the individual spectrum of each fluorescent protein (Clover-AZIN and AZ-mRuby2) from the spectrum of the mixture of the two and adding back the spectrum of a buffer-only blank. (**C**) AZ-mRuby2 [100 pM-1 µM] was titrated against Clover-AZIN [50 nM]. The data was fit to a non-linear regression model to determine the Kd of the protein-protein interaction.

To identify novel small molecule AZIN interactors and to better analyze the intracellular association and localization of antizyme and AZIN in live cells, we designed an intermolecular FRET reporter that uses the Clover and mRuby2 fluorescent proteins as a donor-acceptor pair, respectively. The Clover-mRuby2 FRET pair features a high quantum yield, high extinction coefficient, and overlap between donor emission and acceptor absorbance, making the pair well-suited for intermolecular FRET intensity measurements^13^ (though other pairs may be better for *in vivo* FRET lifetime measurements^14^). To determine the optimal location of each fluorophore in relation to each fusion protein, we tested 4 combinations of N and C terminal fusion proteins (Fig. 1). The most efficient FRET protein-fluorophore combination consisted of an AZIN protein with an N-terminal Clover tag and an antizyme protein with a C-terminal mRuby2 fluorescent tag (Fig. 1A). To validate the FRET sensor performance, we measured the emission spectrum of equimolar concentrations of Clover-AZIN and antizyme-mRuby2 using the donor excitation wavelength (485 nm). This produced FRET-induced changes in the emission spectrum; when compared to the sum of the Clover-AZIN and antizyme-mRuby2 spectra, there was a decrease at the donor emission maximum (515 nm) and an increase at the acceptor emission maximum (600 nm). No such difference was seen when the mRuby2 tag was cleaved from the antizyme protein using the site-specific protease HRV3CP (Fig. 1B).

**Figure 2:**
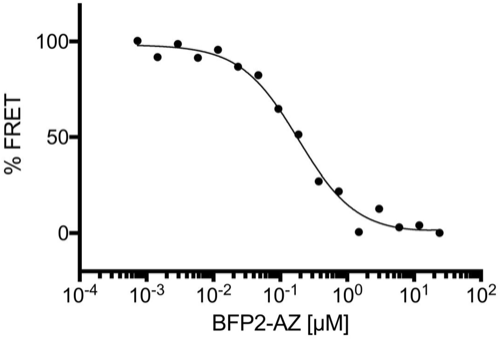
Measuring the Kd of the AZIN-antizyme (AZ) interaction by competition using fluorescent protein fusions. Clover-AZIN [1 µM] and AZ-mRuby2 [1 µM] were incubated to establish equilibrium. BFP2-AZ [48 nM-25 µM] was titrated to compete with AZ-mRuby2 for Clover-AZIN binding and the resulting FRET ratio was plotted.

To probe the K_d_ of the FRET sensor, a constant Clover-AZIN concentration [50 nM] and a range of antizyme-mRuby2 concentrations [1 µM-1 pM] were allowed to equilibrate and the resulting FRET ratio was plotted against the concentration of antizyme-mRuby2 (Fig. 1C). The calculated K_d_ for the Clover-AZIN - antizyme-mRuby2 pair was 22 nM, which is consistent with the 20 nM value measured by ultra-centrifugation^15^. Similar results were obtained when we used BFP-AZIN to compete with constant amounts of Clover-AZIN and antizyme-mRuby2 (Fig. 2). Together, these data demonstrate that the presence of the fluorescent proteins does not affect intermolecular binding affinity and establish the utility of this fusion protein pair as a FRET-based intermolecular interaction sensor.

**Figure 3:**
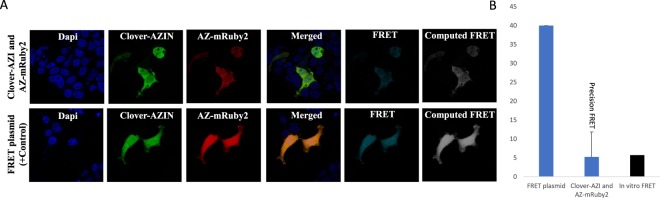
Interaction between Clover-AZIN and AZ-mRuby2 detected by confocal microscopy. (**A**) Following transfection with Clover-AZIN and AZ-mRuby2 (top) or an intramolecular FRET fusion protein control (bottom), images were acquired for DAPI (Ex405 Em474), Clover-AZIN (Ex488 Em513), AZ-mRuby2 (Ex561 Em647), and FRET (Ex488 Em647) using standardized intensity and gain settings. The ImageJ Plug-in PixFRET was used to compute the sensitized-emission FRET signal between Clover-AZIN and AZ-mRuby2 pixel by pixel. Bleedthrough and direct excitation were measured using cells transfected with individual donor and acceptor plasmids and then eliminated from the FRET data, pixel by pixel, to obtain the true (or precision) FRET signal (Computed FRET). **B**) *In vivo* FRET efficiency. Using the calculated signal for the positive-control FRET plasmid as the standard (set to the *in vivo* FRET ratio of 40%), we calculated pixel by pixel FRET efficiency for Clover-AZIN and AZ-mRuby2 to be 7.4%. Maximum FRET efficiency for this protein pair (black) was estimated by multiplying the *in vitro* donor quenching (17%) by the quantum yield of mRuby2 (0.38), yielding 6.5%.

We next overexpressed Clover-AZIN in cells and found that its localization is consistent with its endogens localization (Fig. S.1). Then we examined the interaction between Clover-AZIN and antizyme-mRuby2 via *in vivo* FRET. Energy transfer from the donor to the acceptor was measured and compared to that in a Clover-mRuby2 fusion protein (Figs 3A,B). We found that 7.4% of the energy transferred from the donor Clover-AZIN to the acceptor antizyme-mRuby2 *in vivo*. That is approximately the same as the observed *in vitro* FRET which had 6.5% energy transfer efficiency, suggesting that a similar fraction of antizyme-mRuby2 is bound to Clover-AZIN in cells as *in vitro*. This finding demonstrates that a direct, undisturbed interaction between Clover-AZIN and antizyme-mRuby2 occurs within cells and is not impeded by the intercellular milieu.

### **Mapping** the antizyme:AZIN binding interface

**Figure 4:**
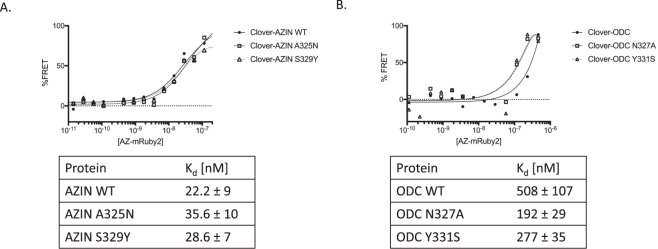
The contribution of conserved amino acid residues to the affinity of antizyme for antizyme inhibitor or ornithine decarboxylase. Point mutations were introduced into the (**A**) AZIN- or (**B**) ODC-Clover fusion proteins. Clover-containing proteins [50 nM] were incubated with a titration of AZ-mRuby2 and the FRET ratio was plotted to determine the Kd of the protein-protein interaction. Kd values are +/− standard error.

AZIN and ODC are highly homologous, yet the binding of AZIN to antizyme is tighter than that of ODC to antizyme, allowing AZIN to counter the growth-suppressing activity of antizyme. A structure of the AZIN-antizyme complex was recently proposed through a combination of modeling techniques including referencing high-resolution structures to achieve reasonable statistics and stereochemistry^16^. Interestingly, the overall binding orientation and key amino acid residues responsible for the binding interaction were found to be either identical or conserved between the antizyme-ODC and AZIN-antizyme protein-protein interactions across all vertebrates, with the exception that ODC-N327 and Y331 are replaced by A325 and S329 in the AZIN protein, respectively. To better understand the key interactions that must be accounted for in AZIN inhibitor design and to confirm the contribution of A325 and S329 to AZIN affinity for antizyme (Fig. 4A), we measured the affinity of ODC-N327A and ODC-Y331S for antizyme (Fig. 4B). These mutations increased the binding affinity from 508 nM to 192 nM and 277 nM. When we introduced A325N and S329Y point mutations into the Clover-AZIN fusion protein, making it more similar to ODC, and measured the resulting binding affinity with antizyme (Fig. 4B), we observed a decrease in affinity and binding energy, albeit not as large. Together, these data support the conclusion that these two key amino acids make a major contribution to the decreased binding affinity of ODC for antizyme relative to AZIN, but that additional differences also play an important role in the increased affinity of AZIN for antizyme. Importantly, our results indicate that small molecules that interact with A325 and/or S329 are likely to be especially potent inhibitors of the antizyme-AZIN interaction that would reduce cell proliferation and decrease tumorigenesis in tumor cells that overexpress either wild-type or edited AZIN.

**Figure 5:**
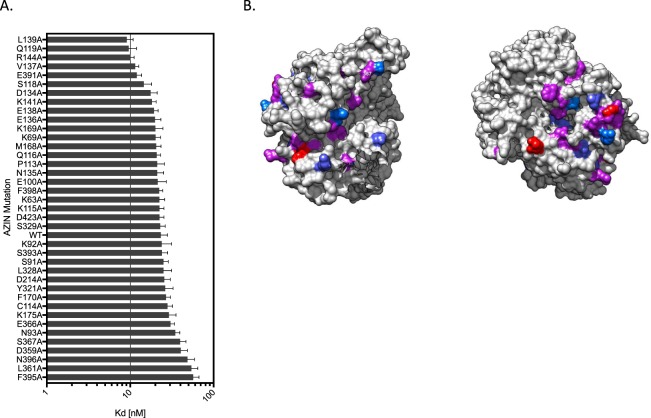
Mutational analysis of antizyme-AZIN binding. (**A**) The affinity of 39 single alanine mutants in AZIN for antizyme were measured using the Clover-AZIN: antizyme-mRubv2 FRET sensor. (**B**) Heatmap of AZIN structure. Red indicates positions where a substitution from the naturally occurring amino acid to an alanine increased affinity; blue indicates a decrease in affinity; purple indicates a marginal effect.

We next analyzed the antizyme-AZIN co-crystal structure to predict potential key residues as targets for small molecule inhibitors. To test these predictions, we employed an alanine screening strategy by introducing 39 alanine point mutations into AZIN positions that were predicted to contribute to the antizyme-AZIN binding interaction (Fig. 5A,B). The distribution of altered affinities suggests that the no single interaction is uniquely important for stabilizing the AZIN-antizyme protein-protein complex. Rather, it is more likely that a large number of small contributions combine to govern the binding affinity. Alanine substitutions closer to the C terminus, between position 325 and 400 were much more likely to weaken the binding interaction, suggesting residues within this region of the protein contribute significantly to the overall binding energy. Conversely, alanine substitutions closer to the beginning of the primary sequence of predicted binding residues (100–200) tended to strengthen the overall binding affinity, suggesting interactions contributed by these regions may modulate the binding interaction and maintain a K_d_ that allows the interaction to remain dynamic and sensitive to intracellular changes in protein concentration. This new analysis of the antizyme-AZIN binding interface suggests that a small molecule inhibitor could interrupt this binding interaction and prevent the intracellular sequestration of antizyme by AZIN, leading to novel therapies for cancer and other proliferative diseases.

## Discussion

Antizyme acts to facilitate ubiquitin-independent degradation of several growth promoting proteins. One antizyme target, ODC, is both an oncogene and the rate-limiting enzyme in polyamine synthesis. Other ODC inhibitors have exhibited anticancer activity in clinical trials, but their clinical success has been modest, partly due to a compensatory activation of polyamine uptake. Antizyme degrades ODC and blocks polyamine uptake but also facilitates degradation of several important growth-promoting molecules, including cyclin D1, SMAD1, and Aurora A kinase. This unique multi-target ability makes antizyme a potent tumor suppressor and cell proliferation inhibitor^1^. AZIN, a catalytically inactive ODC homolog, binds with higher affinity to antizyme and blocks all known antizyme functions.

AZIN retains an analogous pocket which overlaps with the antizyme binding site. A small molecule that binds this pocket should compete with antizyme, thereby increasing available antizyme in the cell, leading to tumor suppression. Despite increasing recognition of the role of AZIN in cancer, no small molecule AZIN antagonists or assays for their development currently exist. The FRET assay that we have developed will identify molecules that inhibit antizyme-AZIN binding. These molecules are expected to release antizyme to inhibit cancer cell growth. The assay has been optimized and adapted for use in the molecular screening of small molecule libraries.

In this assay, clover and mRuby2 florescent proteins have been used as a donor-acceptor pair. The fluorescent fusion proteins exhibit the same affinity for each other as unlabeled antizyme and AZIN and provide a novel assay platform for investigating antizyme:AZIN interaction. Importantly, the intracellular localization of AZIN is consistent with that of unlabeled protein). Furthermore, the fusion proteins exhibit FRET *in vivo*, indicating that the constructs can also measure biologically relevant interactions in live cells. Once the antizyme and AZIN FRET reagents had been validated, we used site-directed mutagenesis to introduce point mutations in AZIN to identify key residues governing the antizyme-AZIN interaction. Unexpectedly, we did not find any single alanine substitution that could have considerable effect on the affinity. Importantly, not all mutations have modest effects; the change of two key conserved residues in ODC for their AZIN counterparts have substantial effects on affinity of antizyme, rendering ODC more like AZIN.

As outlined above, the tumor suppressive effects of antizyme extend beyond antagonizing ODC-mediated synthesis of polyamines and include the regulation of polyamine uptake across the cell membrane and degradation of proteins that are key mediators of cell cycle progression and proliferation (cyclin D1, Mps1, and Aurora A kinase). Thus, AZIN antagonists that successfully compete for antizyme binding, and result in higher free antizyme concentrations have the potential to affect multiple key pathways involved in tumorigenesis. The development of the *in vitro* and *in vivo* FRET assays outlined in this report will improve our ability to perform functional screens for small molecules and natural products that block the antizyme-AZIN interaction. Furthermore, our data regarding the relative importance of various amino-acid sidechains for antizyme-AZIN binding should enable rational optimization of initial lead compounds into high-affinity binders. These results support the development of novel compounds that can specifically target dividing cells and thereby can be used in cancer treatment where the tumor suppressive activity of antizyme is limited by increased AZIN activity.

## Materials and Methods

### Cell culture

HEK293 human embryonic kidney cells lines were purchased from ATCC (Manassas, VA). Cells were grown in DMEM (Gibco, 11885); plus 10% FBS, penicillin-streptomycin, and 1mM l-glutamine.

### Confocal microscopy

Cells were fixed with 4% formaldehyde for 20 minutes and analyzed with a Zeiss LSM 880 META confocal laser scanning microscope (Zeiss, Oberkochen, Germany) equipped with a 63x and 40x Plan-A oil-immersion lenes, using ZEN imaging software in multi-track mode, with the following detection settings.

**Table:** 

	DAPI	Clover	mRuby2	FRET
Laser Wavelength	405 nm	488 nm	561 nm	488 nm
Laser Power	0.82%	1.00%	1.00%	1.00%
Excitation Wavelength	405	488	561	488
Emission Wavelength	474	513	647	647
Detection Bandwidth	54	45	138	138
Detection Wavelength	447–501	490–535	578–716	578–716

### Protein expression and purification

Bacterial expression vectors Clover-pBAD^13^, mRuby2-pBAD^13^, and pBAD-mTAG-BFP2 encoding 6 × -HIS tagged fluorescent proteins with a TEV protease cleavage site were gifts from Michael Davidson (Addgene plasmid # 54575, 54771, 54572; http://n2t.net/addgene:54575, http://n2t.net/addgene:54771, http://n2t.net/addgene:54572; RRID:Addgene_54575, RRID:Addgene_54771, RRID:Addgene_54572). The human *OAZ1* gene was codon optimized for expression in *E. coli* (https://www.idtdna.com/CodonOpt) and synthesized *de novo* (ThermoFisher). Genes encoding N-terminal fluorescent fusion proteins of h*AZIN1*, h*ODC1*, and *E. coli* expression optimized h*OAZI* were synthesized by overlap extension PCR using previously described methods^17^. Expression vectors containing C-terminal fluorescent fusion proteins were synthesized using NEBuilder HiFI DNA Assembly (New England Biolabs). Point mutations in the h*AZIN1* and h*ODC1* genes were achieved using PCR site-directed mutagenesis with Pfu Ultra High-Fidelity DNA polymerase (Agilent) using described protocols. Fusion proteins were espressed and purified according to our previously described methods^18^.

To generate mammalian expression vectors we amplified the full-length cDNA encoding Clover-Antizyme by PCR with primers (F: 5′- cgcGCTAGCcGccATGgTGAAATCCTCCCTGCAGcg-3′; R: 5′-cgcAAGCTTCTTACTTGTACAGCTCGTCCATCC-3′) and for Antizyme-mRuby2 (F: 5′- cgcGCTAGCcGccATGgTGAGCAAGGGCGAG-3′; R: 5′-cgcAAGCTTTTATGCTTCAGCGGAAAAGCTGTC-3′) from pBAD expression vectors above. Subsequently, the purified products were ligated to pcDNA3.1 + (ThermoFisher, MA, USA) according to the manufacturer’s instructions. Plasmid, pcDNA3.1-Clover-mRuby2 was a gift from Kurt Beam (Addgene plasmid # 49089; http://n2t.net/addgene:49089; RRID:Addgene_49089).

All sequences were verified by multiple Sanger sequencing reactions using forward and reverse primers (Eton Biosciences) and analyzed by 4peaks software. Gene, primer, and vector sequences can be found in supplemental information.

### FRET Assay

Purified recombinant protein stocks in 50% glycerol were diluted with TBS (pH 7.4) + 0.15% Tween-20 to 2X final working concentration (100 nM for Clover containing donor proteins and 2 µM mRuby2 acceptor proteins) and transferred to a 96-well plate (Corning 3821). Specifically, acceptor protein or diluent control was transferred into a low-volume 384 well plate (CoStar 3356) in triplicate and serially diluted. Plates were equilibrated at room temp for 1 hour before reading.

Fluorescence was measured using an EnVision plate reader (Perkin Elmer) with a 470/40 excitation filter and either a 515/30 or 600/8 emission filter to measure Clover or mRuby-2 fluorescence, respectively. Curve fitting on donor fluorescence (quenching) was performed in Graphpad Prism v. 7, using global fitting for maximum and minimum fluorescence intensity, constraining the Hill coefficient to 1, and fitting the IC50. Similar IC50s were obtained when fitting was performed on acceptor sensitization after excluding acceptor concentrations above 100 nM. In our *in vitro* FRET determination for AZIN and antizyme, we observed 17% donor quenching and the quantum yield for mRuby2 is 0.38, predicting a 6.4% acceptor sensitization.

### Plasmid transfection

Cells were transfected for 24 h with 2.5 µg plasmid per 60 mm dish using pcDNA3.1^+^-antizyme-mRuby2 and/or pcDNA3.1^+^-Clover-AZIN or pcDNA3.1-Clover-mRuby2 using Lipofectamine^R^ 3000 according to the manufacturer’s instructions (Invitrogen, Carlsbad, CA, USA).

### *In vivo* FRET

The cells were transfected (as described in: plasmid transfection) and fixed cells were imaged by confocal microscopy (as described in: confocal microscopy). PixFRET^19^, an ImageJ plug-in was used for FRET intensity calculations. FRET intensity was measured on a pixel-by-pixel basis using the excitation and emission wavelengths described in “confocal imaging” above. Emission intensity for three imaging conditions was used: A; intensity of the donor channel (excitation and emission for the donor), B; intensity of the acceptor channel (excitation and emission for the acceptor) and C; intensity of the FRET channel (excitation of the donor and emission for the acceptor). Spectral bleed-through (light detected in the FRET channel not due energy transfer) was obtained using cells expressing mRuby2 or Clover alone. To assess the FRET efficiency, sensitization of the FRET plasmid was assumed to have the reported value of 40%^13^. This gave a calculated Pixel by pixel FRET for Clover-AZIN and antizyme-mRuby2 of 7.38% (30 control cells and 32 Clover-AZIN and antizyme-mRuby2 overexpressed cells).

## Supplementary information

Supplement
