# Supplementary material for: Developing a novel FRET assay, targeting the binding between Antizyme-AZIN
Source: Sci Rep. 2019 Mar 15;9:4632. doi: 10.1038/s41598-019-40929-4 (PMC6420652; doi:10.1038/s41598-019-40929-4)
Supplement: Supplementary file 1 — Supplement [file 41598_2019_40929_MOESM1_ESM.pdf]

Developing a novel FRET assay, targeting the binding between Antizyme-AZIN.

Aram Ghalali[1 ¶], James M. Rice[1, #a ¶], Amanda Kusztos[1, #b], Finith Jernigan[2,#a], Bruce R. Zetter[1] and Michael S. Rogers[1*]

Supplementary Figure and Tables

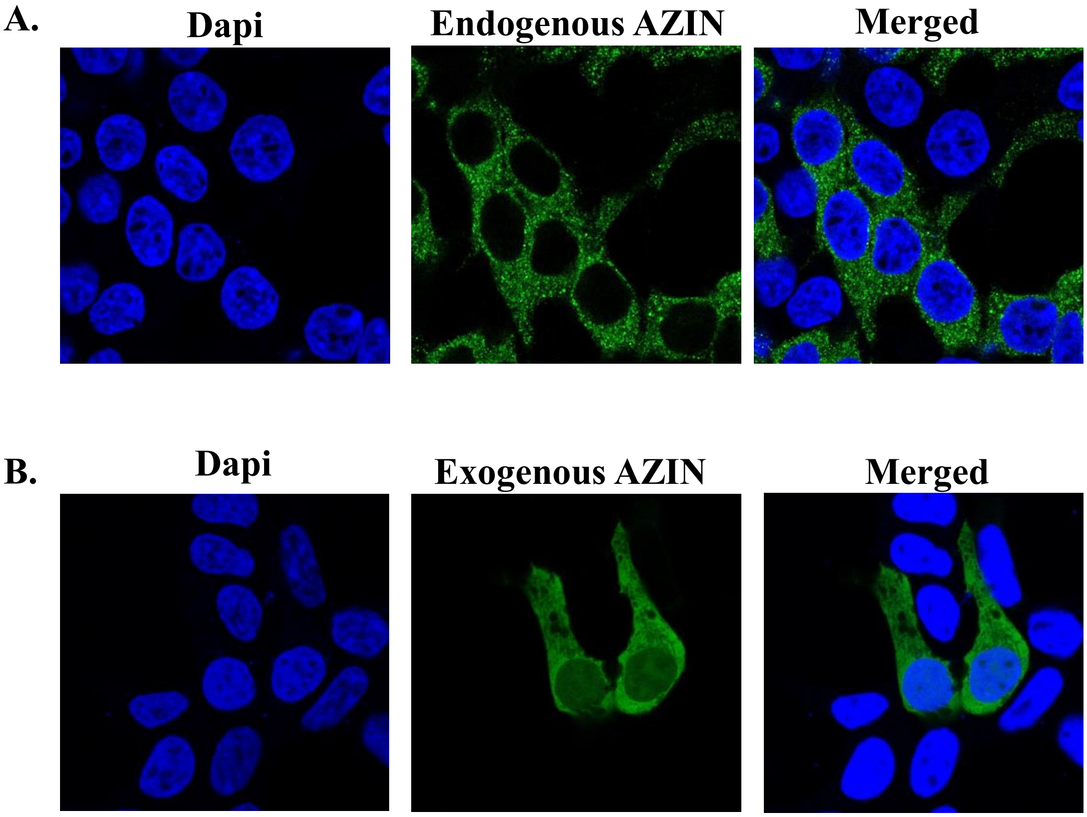

Figure S.1. Endogenous and exogenous AZIN is localized at the cytoplasm. (A), Human Embryonic Kidney (HEK293) cells were stained for AZIN or (B), transfected with 2.5µg of pcDNA-Clover-AZIN plasmid for 24h and analyzed by confocal microscopy.

Supplemental Table 1. AZIN Mutant Primer Sequences

AZIN Mutation	Direction	Primer Sequence 5'-->3'
K92A	Forward	GGATTTGCTTGTTCCAGTGCAAATGAAATGGCTTTAG
	Reverse	CTAAAGCCATTTCATTTGCACTGGAACAAGCAAATCC
K141A	Forward	CAATGAAATTGAATTGAAGGCAATTGCACGTAATCACCC
	Reverse	GGGTGATTACGTGCAATTGCCTTCAATTCAATTTCATTG
Y321A	Forward	GTATTATATGAATGATGGTGTTGCTGGTTCTTTTGCAAGTAAACTG
	Reverse	CAGTTTACTTGCAAAAGAACCAGCAACACCATCATTCATATAATAC
F398A	Forward	CTGCTTTTAATGATGCTCAGAGGCCAGCC
	Reverse	GGCTGGCCTCTGAGCATCATTAAAAGCAG
F395A	Forward	CCATGAACCATCTGCTGCTAATGATTTTCAGAGGC
	Reverse	GCCTCTGAAAATCATTAGCAGCAGATGGTTCATGG
E100A	Forward	GCTTTAGTGCAAGCGTTGGGTGTACCTC
	Reverse	GAGGTACACCCAACGCTTGCACTAAAGC
K69A	Forward	CATTCTACACAGTGGCGTGCAACTCTGCTCC
	Reverse	GGAGCAGAGTTGCACGCCACTGTGTAGAATG
K169A	Forward	GAAGAGGGTAACATGGCGTTTGGCACTACCC
	Reverse	GGGTAGTGCCAAACGCCATGTTACCCTCTTC
K175A	Forward	GTTTGGCACTACCCTGGCGAACTGTAGGCATCTC
	Reverse	GAGATGCCTACAGTTCGCCAGGGTAGTGCCAAAC
L361A	Forward	GTCCATCCTGTGATGAGGCTGATCAAATTGTGGAAAG
	Reverse	CTTTCCACAATTTGATCAGCCTCATCACAGGATGGAC
F170A	Forward	GAGGGTAACATGAAGGCTGGCACTACCCTGAAG
	Reverse	CTTCAGGGTAGTGCCAGCCTTCATGTTACCCTC
I137A	Forward	CCTGACATGTGACAATGAAGCTGAATTGAAGAAAATTGC
	Reverse	GCAATTTTCTTCAATTCAGCTTCATTGTCACATGTCAGG

R144A	Forward	GAATTGAAGAAAATTGCAGCTAATCACCCAAATGCCAAG
	Reverse	CTTGGCATTTGGGTGATTAGCTGCAATTTTCTTCAATTC
E204A	Forward	GTTTCGAGTGCTTGCAAAGCATCTCAAGTATATGTACATG
	Reverse	CATGTACATATACTTGAGATGCTTTGCAAGCACTCGAAAC
D134A	Forward	GTGAATATCCTGACATGTGCCAATGAAATTGAATTGAAG
	Reverse	CTTCAATTCAATTTCATTGGCACATGTCAGGATATTCAC
E366A	Forward	CTTGATCAAATTGTGGCAAGCTGTCTTCTTCCTG
	Reverse	CAGGAAGAAGACAGCTTGCCACAATTTGATCAAG
E391A	Forward	GCAGATTCTTTCCATGCACCATCTGCTTTTAATG
	Reverse	CATTAAAAGCAGATGGTGCATGGAAAGAATCTGC
D214A	Forward	GTACATGCTCTATCTGCTGCTCGATGTGTGTTTG
	Reverse	CAAACACACATCGAGCAGCAGATAGAGCATGTAC
D423A	Forward	GCTGGAATTACTTCAGCCTCAATGATGAAGAAC
	Reverse	GTTCTTCATCATTGAGGCTGAAGTAATTCCAGC
K63A	Forward	GTAGTGGCTCAGATAGCGCCATTCTACACAGTG
	Reverse	CACTGTGTAGAATGGCGCTATCTGAGCCACTAC
K115A	Forward	CATAAGTCCTTGCGCGCAAGTGTCTCAG
	Reverse	CTGAGACACTTGCGCGCAAGGACTTATG
A123S	Forward	GTCTCAGATAAAGTATAGAGCAAAAGTTGGAGTG
	Reverse	CACTCCAACTTTTGCTCTATACTTTATCTGAGAC
E138A	Forward	CATGTGACAATGAAATTGCATTGAAGAAAATTGCACG
	Reverse	CGTGCAATTTTCTTCAATGCAATTTCATTGTCACATG
L139A	Forward	CATGTGACAATGAAATTGAAGCGAAGAAAATTGCACGTAATC
	Reverse	GATTACGTGCAATTTTCTTCGCTTCAATTTCATTGTCACATG
L328A	Forward	GGTTCTTTTGCAAGTAAAGCGTCTGAGGACTTAAATACC
	Reverse	GGTATTTAAGTCCTCAGACGCTTTACTTGCAAAAGAACC

S367G	Forward	GATCAAATTGTGGAAGGCTGTCTTCTTCCTG
	Reverse	CAGGAAGAAGACAGCCTTCCACAATTTGATC
S367A	Forward	CTTGATCAAATTGTGGAAGCCTGTCTTCTTCCTGAGC
	Reverse	GCTCAGGAAGAAGACAGGCTTCCACAATTTGATCAAG
S367N	Forward	GATCAAATTGTGGAAAACTGTCTTCTTCCTGAGC
	Reverse	GCTCAGGAAGAAGACAGTTTTCCACAATTTGATC
N327A	Forward	GGCGTCTATGGATCATTTGCTTGCATACTCTATGACC
	Reverse	GGTCATAGAGTATGCAAGCAAATGATCCATAGACGCC
Y331A	Forward	GATCATTTAATTGCATACTCGCTGACCACGCACATGTAAAG
	Reverse	CTTTACATGTGCGTGGTCAGCGAGTATGCAATTAAATGATC
Y331S	Forward	CATTTAATTGCATACTCTCTGACCACGCACATGTAAAG
	Reverse	CTTTACATGTGCGTGGTCAGAGAGTATGCAATTAAATG
S91A	Forward	GAACCGGATTTGCTTGTTCCGCTAAAAATGAAATGGCTTTAG
	Reverse	CTAAAGCCATTTCATTTTTAGCGGAACAAGCAAATCCGGTTC
N93A	Forward	GATTTGCTTGTTCCAGTAAAGCTGAAATGGCTTTAGTGCAAG
	Reverse	CTTGCACTAAAGCCATTTCAGCTTTACTGGAACAAGCAAATC
P113A	Forward	CATTATTTACATAAGTGCTTGCAAGCAAGTGTC
	Reverse	GACACTTGCTTGCAAGCACTTATGTAAATAATG
C114A	Forward	CAGAAAACATTATTTACATAAGTCCTGCCAAGCAAGTGTCTCAGATAAAGTATG
	Reverse	CATACTTTATCTGAGACACTTGCTTGGCAGGACTTATGTAAATAATGTTTTCTG
Q116A	Forward	CATAAGTCCTTGCAAGGCAGTGTCTCAGATAAAG
	Reverse	CTTTATCTGAGACACTGCCTTGCAAGGACTTATG
S118A	Forward	CCTTGCAAGCAAGTGGCTCAGATAAAGTATGC
	Reverse	GCATACTTTATCTGAGCCACTTGCTTGCAAGG
Q119A	Forward	GTCCTTGCAAGCAAGTGTCTGCGATAAAGTATGCAGCAAAAG
	Reverse	CTTTTGCTGCATACTTTATCGCAGACACTTGCTTGCAAGGAC

N135A	Forward	GAGTGAATATCCTGACATGTGACGCTGAAATTGAATTGAAGAAAATTG
	Reverse	CAATTTTCTTCAATTCAATTTCAGCGTCACATGTCAGGATATTCACTC
E136A	Forward	GAATATCCTGACATGTGACAATGCAATTGAATTGAAGAAAATTGCACG
	Reverse	CGTGCAATTTTCTTCAATTCAATTGCATTGTCACATGTCAGGATATTC
M168A	Forward	GAGGTGAAGAGGGTAACGCGAAGTTTGGCACTACC
	Reverse	GGTAGTGCCAAACTTCGCGTTACCCTCTTCACCTC
A325N	Forward	GGTGTTTATGGTTCTTTTAATAGTAAACTGTCTGAGGAC
	Reverse	GTCCTCAGACAGTTTACTATTAAAAGAACCATAAACACC
S329A	Forward	CTTTTGCAAGTAAACTGGCTGAGGACTTAAATACC
	Reverse	GGTATTTAAGTCCTCAGCCAGTTTACTTGCAAAAG
S329Y	Forward	GGTTCTTTTGCAAGTAAACTGTATGAGGACTTAAATACCATTCC
	Reverse	GGAATGGTATTTAAGTCCTCATACAGTTTACTTGCAAAAGAACC
D359A	Forward	CTTTGGGGTCCATCCTGTGCTGAGCTTGATCAAATTGTG
	Reverse	CACAATTTGATCAAGCTCAGCACAGGATGGACCCCAAAG
S393A	Forward	GATTCTTTCCATGAACCAGCTGCTTTTAATGATTTTC
	Reverse	GAAAATCATTAAAAGCAGCTGGTTCATGGAAAGAATC
N396A	Forward	CATGAACCATCTGCTTTTGCTGATTTTCAGAGGCCAGCC
	Reverse	GGCTGGCCTCTGAAAATCAGCAAAAGCAGATGGTTCATG

Supplemental Table 2. N-Terminal Fluorescent Protein Primer Sequences

N-Terminal Fluorescent Fusion Protein	Direction	Primer Sequence 5' --> 3'
6x His-Clover-Thr-AZIN	Forward	CCAAGCTTCGAATTCTTAT TAAGCTTCAGCGGAAAA
	Reverse	TGGACGAGCTGTACAAG CTGGTTCCGCGTGGTAGT AAAGGATTTATTGATGATGC
6x His-mRuby2-HRV3CP-AZ	Forward	G CCA AGC TTC GAA TTC TTA CTA CTC CTC CTC CTC TCC
	Reverse	ATG GAC GAG CTG TAC AAG CTT GAA GTC CTC TTT CAG GGA CCC gtg aaa tcc tcc ctg cag c
6x His-mTag-BFP2-HRV3CP-mTagBFP2	Forward	CCAAGCTTCGAATTCTTA CTA CTC CTC CTC CTC TCC
	Reverse	CTGGGGCACAAGCTTAAT CTT GAA GTC CTC TTT CAG GGA CCC gtg aaa tcc tcc ctg cagc
6x-His-Clover-Thr-ODC	Forward	CCAAGCTTCGAATTCTTAT CTACACATTAATACTAGCC
	Reverse	TGGACGAGCTGTACAAG CTGGTTCCGCGTGGTAGT AACAACTTTGGTAATGAAGA

Supplemental Table 3. C-Terminal Fluorescent Protein Primer Sequences

C-Terminal Fluorescent Fusion Protein	Direction	Primer Sequence 5' --> 3'
6x His-AZIN-Thr-Clover	Forward	ctgtacttccagggcATGAAAGGATTTATTGATGATGCAAAC
	Reverse	ccttgctcaccatgcgactaccacgcggaaccagcttAGCTTCAGCGGAAAAGCTG
6x His-AZ-HRV3CP-mRuby2	Forward	ctgtacttccagggcGTGAAATCCTCCCTGCAG
	Reverse	cccttagacaccatgccgggtccctgaaagaggacttcaagcTCCTCCTCCTCTCCCGAA
6x His-AZ-HRV3CP-mTagBFP2	Forward	ctgtacttccagggcGTGAAATCCTCCCTGCAG
	Reverse	cccttagacaccatgccgggtccctgaaagaggacttcaagCTCCTCCTCTCCCGAAGAC
